# Unilateral axillary lymph node fluorodeoxyglucose uptakes after coronavirus disease 2019 vaccination

**DOI:** 10.22038/AOJNMB.2021.59883.1419

**Published:** 2022

**Authors:** Tomonori Chikasue, Seiji Kurata, Akiko Sumi, Akihiro Matsuda, Fumihiro Tsubaki, Kiminori Fujimoto, Toshi Abe

**Affiliations:** Department of Radiology, Kurume University School of Medicine, Japan

**Keywords:** COVID-19, Vaccination, PET/CT, Axillary lymph nodes, ^ 18^F-FDG uptake

## Abstract

Vaccination against coronavirus disease 2019 (COVID-19) started in early December 2020 worldwide, and healthcare workers in Japan were vaccinated in February 2021. We encountered three patients who underwent ^18^F-fluorodeoxyglucose (FDG) positron emission tomography/computed tomography (PET/CT) for cancer screening at our institution, showing FDG uptakes in the axillary lymph nodes, which seemed to be reactive changes. Two of them were males in their 40s and one was a female in her 50s; all of them were healthcare workers. The medical history revealed that they received the Pfizer-BioNTech COVID-19 vaccination twice at their left shoulders before the FDG PET/CT examination. The degree of FDG uptakes were maximum standardized uptake value (SUV_max_)=3.2–9.9, SUV_max_=5.9–10.3, and SUV_max_=2.8–7.9, respectively. They were diagnosed with reactive lymph nodes because of vaccination owing to the absence of abnormal FDG PET/CT findings at other sites. As COVID-19 vaccination becomes more widespread in Japan, radiologists should be aware of these findings to avoid misdiagnosis of FDG uptakes in pathological lymph nodes and to prevent unnecessary additional examinations. Recently, similar FDG PET/CT findings have been reported after receiving the COVID-19 vaccination, and we will report it with a literature review.

## Introduction

 The coronavirus disease 2019 (COVID-19) has been reported since the end of 2019 and quickly spread worldwide, causing a pandemic. Vaccination against COVID-19 has been performed in various countries, starting in the UK at the end of 2020, and vaccination of healthcare workers in Japan started in February 2021. At this time, ^18^F-fluorodeoxyglucose (FDG) uptakes in reactive lymph nodes were observed in patients who underwent positron emission tomography/computed tomography (PET/CT) after the COVID-19 vaccination. 

 When the vaccination history is unclear, such an FDG uptake may be difficult to distinguish from the FDG uptake in diseased lymph nodes. Vaccination is expected to expand further in the future, and many similar cases likely occur. To avoid unnecessary imaging studies, we will describe our experience.

## Case Report

***Case 1***

 A 40-year-old male healthcare worker of our hospital had received the second dose of COVID-19 vaccine in the left deltoid muscle 6 days earlier and had no specific medical history. The patient was aware of pain at the injection site and had no other adverse reactions to the vaccine. FDG PET/CT was performed for cancer screening. The imaging findings revealed multiple FDG uptakes in six left axillary lymph nodes from levels 1 to 3 (maximum standardized uptake value [SUV_max_]=3.2–9.9). The levels of the axillary lymph nodes are based on the 8^th^ edition of the UICC TNM Classification (1). 

**Figure 1:**
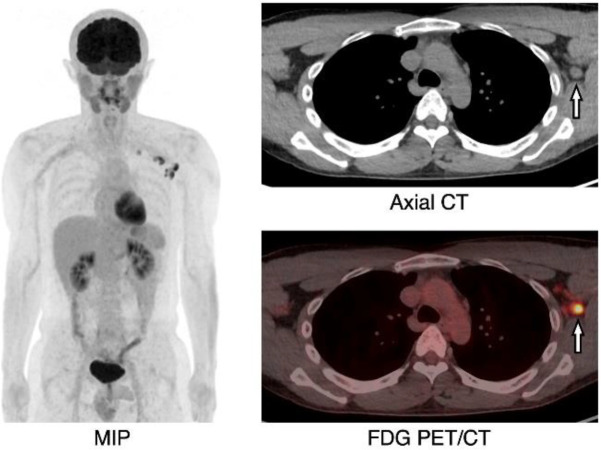
A 40-year-old male, FDG PET/CT (MIP: maximum intensity projection) showed multiple FDG uptakes in six left axillary lymph nodes from levels 1 to 3 (SUV_max_=3.2–9.9). Axial CT and FDG PET/CT show the largest lymph node with the minimum diameter of >10 mm (arrow, short axis: 12 mm, SUV_max_=9.9)

Three lymph nodes with a maximum short axis of >10 mm were found (up to 12 mm) (Figure 1).

***Case 2***

**Figure 2:**
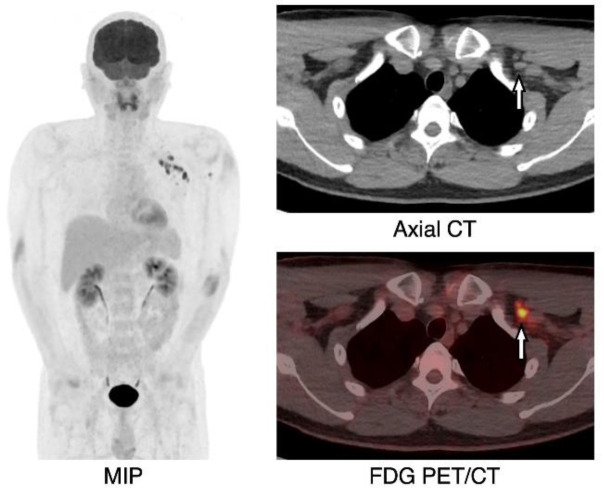
A 49-year-old male, FDG PET/CT (MIP) showed multiple FDG uptakes in 12 left axillary lymph nodes from levels 1 to 3 (SUV_max_=5.9–10.3). Axial CT and FDG PET/CT showed the lymph nodes that were the largest in this case (arrow, short axis: 6 mm, SUV_max_=10.3)

 A 49-year-old male healthcare worker of our hospital being treated for juvenile hyper-tension, obesity, and glucose intolerance (blood glucose levels are controlled by diet alone) had received a second dose of COVID-19 vaccine in his left deltoid 3 days earlier. He was aware of pain at the injection site and had no other adverse reactions to the vaccine. FDG PET/CT was performed for cancer screening. FDG uptake was found in 12 left axillary lymph nodes from level 1 to 3 (SUV_max_=5.9–10.3). No lymph nodes with the maximum short axis of >10 mm were found (up to 6 mm) (Figure 2).

***Case 3***

**Figure 3:**
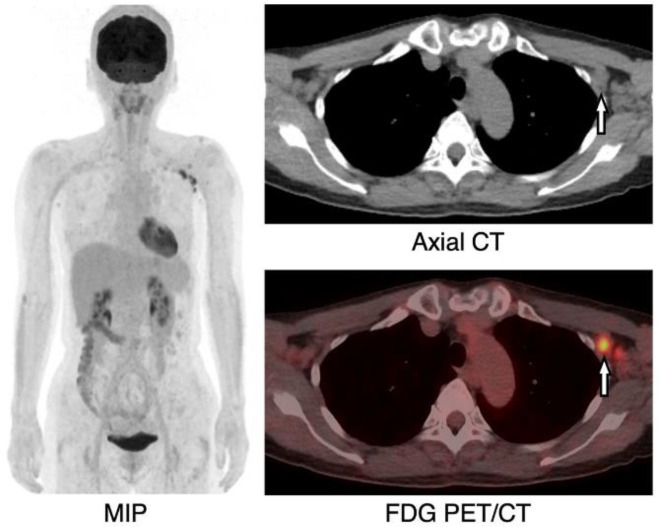
A 55-year-old female, FDG PET/CT (MIP) showed multiple FDG uptakes in eight left axillary lymph nodes from levels 1 to 3 (SUV_max_=2.8–7.9). Axial CT and FDG PET/CT showed the largest of the lymph nodes with the minimum diameter of >10 mm (arrow, short axis: 11 mm, SUV_max_=7.9)

 A 55-year-old female healthcare worker from another hospital had received a second dose of COVID-19 vaccine in her left deltoid 12 days earlier. The patient experienced pain at the injection site and a fever of 38℃. No other adverse reactions related to the vaccine were observed. FDG PET/CT was performed for cancer screening. The patient had no relevant medical history (including breast cancer). However, FDG uptake was found in eight left axillary lymph nodes from levels 1 to 2 (SUV_max_=2.8–7.9). A lymph node with a maximum short axis of >10 mm was found

 (Up to 11 mm) (Figure 3).

 In all patients, no significant FDG uptake was observed at other sites, and we conclude that FDG uptake in the lymph nodes is associated with COVID-19 vaccination. We determined that no additional testing was needed in all three patients.

 The characteristics of each patient are summarized in Table 1.

**Table 1:** Patient characteristics

	**Case 1**	**Case 2**	**Case 3**
Sex	Male	Male	Female
Age	40	49	55
Interval between vaccination and PET/CT	6 days	3 days	12 days
Level of axillary enlarged LNs	1 to 3	1 to 3	1 to 2
Maximum short-axis diameter of enlarged LN	12 mm	6 mm	11 mm
Number of LNs with >10 mm in the short axis	3	0	1
SUV_max_ of LNs	3.2–9.9	5.9–10.3	2.8–7.9
Number of FDG-avid LNs	6	12	8

**Abbreviations:** LN, lymph node; SUV_max_, the maximum of standardized uptake values

## Discussion

 COVID-19 is pneumonia caused by severe acute respiratory syndrome coronavirus 2 (SARS-CoV-2), which started to spread around December 2019 and caused a pandemic. Vaccination for the newly developed COVID-19 began in the USA and Europe in early December 2020. In Japan, vaccination with Pfizer-BioNTech COVID-19 vaccine was started for healthcare workers in February 2021.

 Adverse effects of the COVID-19 vaccines include enlargement of axillary, supraclavicular, and cervical lymph nodes ipsilateral to the triangular vaccination (2, 3). The distribution of lymphadenopathy has also been reported with seasonal influenza, H1N1 influenza A (4-6), and other vaccines. Inoculation of the deltoid muscle with COVID-19 results in clinically palpable ipsilateral axillary lymphadenopathy in at least 15% of patients after the second inoculation (7).

 Cohen et al. (8) reported that in patients who underwent FDG PET after the COVID-19 vaccination in Israel, 45.8% who received the second dose had vaccine-associated hyper-metabolic lymphadenopathy (VAHL) in the axillary or supraclavicular lymph node ipsilateral at the vaccination site. In the group that received two doses of the vaccine, the percentage of VAHL in axillary lymph nodes was 99.4%, 43.4%, and 13.2% for levels 1, 2, and 3, respectively. In the group, PET/CT demon-strated VAHL with SUV_max_ of 2.76 (range 1.97–4.29). In three patients we managed, two had enlarged axillary lymph nodes at level 3 and one had enlarged axillary lymph nodes at level 2. No enlargement of the supraclavicular or cervical lymph nodes was noted. The SUV_max_ indicated by PET/CT was 3.2–9.9, 5.9–10.3, and 2.8–7.9, respectively, which were higher than those reported above. This may reflect changes due to differences in the equipment used. The size of VAHL has been reported to be up to 13 mm on the short axis (7), and the size in this patient showed the same degree of enlargement.

 Eifer et al. (9) have reported that the incidence of VAHL after COVID-19 vaccination was significantly lower in immunocompromisedand elderly patients. Other reports have also observed a reduced induced immune response to the COVID-19 vaccine in immuno-compromised patients (10, 11). COVID-19 vaccine is a novel mRNA-based vaccine that induces a pronounced germinal center (GC) response in the lymph nodes that respond to the mRNA vaccine. Moreover, the GC response has been found to play an important role in humoral responses (12, 13). Dan Cohen et al. (14) have found that the incidence of VAHL was significantly lower in a group of patients treated with anti-CD20 antibodies and reported that VAHL may reflect B-cell proliferation in GCs at the early stages of humoral response to vaccination. Although all three patients were middle-aged and had normal immune status, lymph node uptakes after COVID-19 vaccination should be carefully interpreted in patients with compromised immunity, such as those undergoing chemotherapy.

 Currently, there are no established practice guidelines or certain opinions on the timing of FDG PET imaging after vaccination. Experiences with influenza and other vaccinations performed to date indicate that vaccine-related lymph node FDG uptake generally occurs within 7 days of vaccination and subsides by 12–14 days (4-6). The FDG uptake in VAHL after COVID-19 vaccination has been reportedly lower in the first 5 days or 3 weeks after the first vaccination and after 20 days after the booster vaccination (8). In our patients, FDG PET performed 12 days after the second vaccination showed FDG uptake in VAHL, which is consistent with their report.

 There is no consensus on the need for follow-up on FDG uptake in lymph nodes after COVID-19 vaccination. Previous reports (15) have suggested that if FDG uptake in the lymph nodes is not an intrinsic disease or metastasis and is considered to be a finding of vaccination based on clinical judgment, no further follow-up is necessary. In our patients, no metastasis or other pathological enlargements of the lymph nodes were observed, and thereby, no further examination was required. When FDG uptake in the lymph nodes indicates the possibility of malignancy or metastasis, lymph node morphology should be evaluated if clinically significant (e.g., impact on staging). If morbid progression is positively suspected, re-evaluation with ultrasound (US) or CT within 2–6 weeks and US-guided biopsy should be suggested if abnormal lymph nodes remain(15,17).

 In this report, we encountered three patients with FDG uptake in the axillary lymph nodes due to the COVID-19 vaccination. It is expected that similar cases will increase in Japan in the future. Some of the lymph nodes with FDG uptake had a short axis of >10 mm, and thus, the COVID-19 vaccination history should be confirmed to differentiate them from pathological lymph node enlargement.
